# The Gene *Transformer* of *Anastrepha* Fruit Flies (Diptera, Tephritidae) and Its Evolution in Insects

**DOI:** 10.1371/journal.pone.0001239

**Published:** 2007-11-28

**Authors:** María Fernanda Ruiz, Andreina Milano, Marco Salvemini, José María Eirín-López, André L. P. Perondini, Denise Selivon, Catello Polito, Giuseppe Saccone, Lucas Sánchez

**Affiliations:** 1 Centro de Investigaciones Biológicas (CSIC), Madrid, Spain; 2 Dipartimento delle Scienze Biologiche–Sezione di Genetica e Biologia Molecolare, Università degli Studi di Napoli “Federico II”, Napoli, Italy; 3 Instituto di Genetica e Biofisica−Adriano Buzzati−Traverso (ABT), Consiglio Nazionale delle Ricerche (CNR), Napoli, Italy; 4 Departamento de Biología Celular y Molecular, Universidade da Coruña, Coruña, Spain; 5 Departamento de Genética e Biologia Evolutiva, Instituto de Biociências, Universidade de São Paulo, Sao Paulo, Brazil; Ecole Normale Supérieure de Lyon, France

## Abstract

In the tephritids *Ceratitis capitata* and *Bactrocera oleae*, the gene *transformer* acts as the memory device for sex determination, via an auto-regulatory function; and functional Tra protein is produced only in females. This paper investigates the evolution of the gene *tra*, which was characterised in twelve tephritid species belonging to the less extensively analysed genus *Anastrepha*. Our study provided the following major conclusions. Firstly, the memory device mechanism used by this gene in sex determination in tephritids likely existed in the common ancestor of the *Ceratitis*, *Bactrocera* and *Anastrepha* phylogenetic lineages. This mechanism would represent the ancestral state with respect to the extant cascade seen in the more evolved *Drosophila* lineage. Secondly, Transformer2-specific binding intronic splicing silencer sites were found in the splicing regulatory region of *transformer* but not in *doublesex* pre-mRNAs in these tephritids. Thus, these sites probably provide the discriminating feature for the putative dual splicing activity of the Tra-Tra2 complex in tephritids. It acts as a splicing activator in *dsx* pre-mRNA splicing (its binding to the female-specific exon promotes the inclusion of this exon into the mature mRNA), and as a splicing inhibitor in *tra* pre-mRNA splicing (its binding to the male-specific exons prevents the inclusion of these exons into the mature mRNA). Further, a highly conserved region was found in the specific amino-terminal region of the tephritid Tra protein that might be involved in Tra auto-regulatory function and hence in its repressive splicing behaviour. Finally, the Tra proteins conserved the SR dipeptides, which are essential for Tra functionality.

## Introduction

Perpetuation by sexual reproduction is the rule within the animal Kingdom. A plethora of sex determination mechanisms exist which commit the embryo to following either the male or female developmental pathway. The mechanism underlying this process has been thoroughly analysed in *Drosophila melanogaster*. In this species, sex determination is under the control of the gene *Sex lethal (Sxl)*. The epistatic relationships between *Sxl* and the other sex determination genes *transformer (tra)*, *transformer-2 (tra-2)* and *doublesex (dsx)* have revealed a hierarchical interaction to exist among them. Their characterisation has shown that the sex-specific splicing of their primary transcripts controls their expression during development, the product of one gene controlling the sex-specific splicing of the pre-mRNA of the downstream gene in the sex determination cascade (reviewed in [1]).

The gene *Sxl*, which is at the top of this cascade, acts as the memory device for female sexual development via its auto-regulatory function: the Sxl protein participates in the female-specific splicing of its own pre-mRNA [2], [3]. The downstream target of *Sxl* is the gene *transformer (tra)*. A transcript found in both males and females encodes a non-functional truncated Tra protein, and a female-specific transcript encodes the functional Tra protein [4], [5], [6], [7], [8]. The Tra product and the product of the constitutive gene *transformer-2 (tra-2)* [9], [10] control the sex-specific splicing of the pre-mRNA of the gene *doublesex (dsx)*, the last gene in the genetic cascade, and which is transcribed in both sexes [11], [12], [13], [14], [15], [16]. In females, the Tra-Tra2 complex directs the splicing of the *dsx* pre-mRNA according to the female mode, giving rise to the female DsxF protein that promotes female sexual development. In males, where no functional Tra protein is available, the *dsx* pre-mRNA follows the default, male mode of splicing, which produces male DsxM protein. This promotes male sexual development.

Sex determination mechanisms have long been of major interest from both developmental and evolutionary points of view. The search in different insects for genes homologous to the sex determination genes of *D. melanogaster* is underway. The aim is to determine how much of the sex determination genetic cascade has been modified between the more ancient dipteran phylogenetic lineages and the drosophilid lineage. The gene *tra* of *Drosophila simulans, D. erecta, D. hydei* and *D. virilis* has been characterised [17] and an unusually high degree of divergence found with that of *D. melanogaster*. Even so, *D. melanogaster tra* mutants can all be rescued by the *tra* of these species [17].

Outside the genus *Drosophila*, *tra* was first characterised in *Ceratitis capitata* [18] and recently in *Bactrocera oleae* [19]. As in *Drosophila*, in *Ceratitis* and *Bactrocera* alternative splicing also regulates the expression of *tra* so that only females contain the full-length protein. The *Ceratitis capitata (Cctra)* and *Bactrocera oleae (Botra) tra* genes have male-specific exons, which contain translation stop codons; their inclusion in the mature mRNA produces truncated and most probably non-functional Tra protein. In females, these exons are spliced out, and a mature mRNA is made that produces a functional Tra protein. Surprisingly, putative Tra-Tra2 binding sites have been found in the male-specific exons of *Cctra* [18] and *Botra* [19]. The injection of the respective *tra-*dsRNA in *Ceratitis* [20] and in *Bactrocera* [19] results in the destruction of endogenous *tra* function in both species and the subsequent male-specific splicing of the endogenous *tra* pre-mRNA, ensuing in the complete transformation of females into fertile XX pseudomales.

The early results in *Ceratitis* led to Pane et al. [18] to propose a novel auto-regulatory function for the *Cctra* gene with respect to its *Drosophila* homologue. In *C. capitata*, the gene *tra* would play the key regulatory role, acting as the memory device for sex determination through its auto-regulatory function. The Tra protein would act as a splicing inhibitor of its own pre-mRNA splicing. This was based on the idea that the Tra-Tra2 complex binds to the Tra-Tra2 binding sites in the *tra* pre-mRNA−because of the sequence conservation of these sites−inhibiting the incorporation of the male-specific exons into the mature *tra* mRNA. This would also apply to the gene *tra* of *B. oleae* [19].

To better analyse the evolution of *tra* gene, in the present work its characterisation was undertaken in tephritid species belonging to the less extensively analysed genus *Anastrepha*. We chose those species which, according to morphological [21] and molecular data [22], belong to distinct intrageneric taxonomic groups. The present analysis therefore included *Anastrepha obliqua, A. ludens, A. amita* and *A. sororcula*, plus the four closely related species of the so-called *Anastrepha fraterculus* complex−*A.* sp.1 *aff*. *fraterculus, A.* sp.2 *aff*. *fraterculus, A.* sp.3 *aff*. *fraterculus* and *A.* sp.4 *aff*. *fraterculus* [23], [24], all of which belong to the *fraterculus* group [21]–along with *A. serpentina, A. striata* and *A. bistrigata* (of the *serpentina* group, see 25]) and *A. grandis* (of the *grandis* group).

The gene *tra* in the reference species *A. obliqua* was first isolated and its molecular organisation, expression pattern and encoded product studied. The *tra* ORFs in the other *Anastrepha* species were then identified, and a comparative analysis of all the known insect Tra proteins undertaken. In this way, a comparison of the Tra protein at different phylogenetic levels was made. Within-genus comparisons of the members of *Drosophila* and *Anastrepha*; distinct genera of the same family, such as *Ceratitis, Bactrocera* and *Anastrepha* (Tephritidae); and two different families of the same order (Diptera), such as Drosophilidae and Tephritidae. Next, the genomic *tra* region that controls the sex-specific splicing of its primary transcript was characterised and compared in *C. capitata*, *B. oleae* and the above *Anastrepha* species. This should indicate whether the molecular organisation of gene *tra* in *Ceratitis* and in *Bactrocera* arose before or after the splitting off of the frugivorous Tephritidae lineage. Finally, the phylogeny of gene *tra* in these different insects was investigated.

## Results

### The molecular organisation of *tra* in *Anastrepha obliqua*, and its product

The strategy followed to determine the molecular organisation of *Anastrepha obliqua tra* gene *(Aotra)* is described in Materials and Methods.

**Figure 1:**
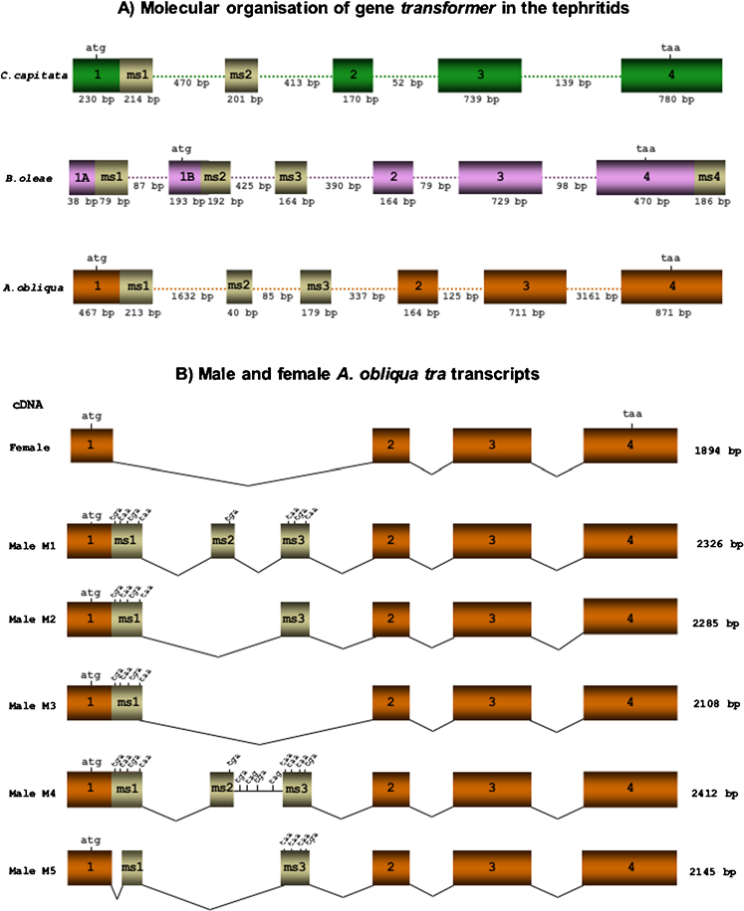
Comparison of the molecular organisation of the gene *tra* of *C. capitata, B. oleae* and *A. obliqua* (A) and the transcripts encoded by the *A. obliqua tra* gene (B). Exons (boxes) and introns (lines) are not drawn to scale. The numbers inside the boxes indicate the number of the exon; ms1, ms2 and ms3 stand for the male-specific exons. The beginning and the end of the ORF are indicated by ATG and TAA respectively. The longest female mRNA is shown. The male-specific transcripts show the stop codons in the mature mRNA; these depend on the male-specific exons incorporated.

Figures 1A and B shows the molecular organisation of the *Aotra* gene. The transcription unit is made up of 7665 bp, and the transcription start site located at –197 bp from the initial ATG of the ORF. It is composed of 4 exons (1–4) common to both sexes, and three male-specific exons, ms1, ms2 and ms3, located between exons 1 and 2. The *Aotra* gene produces three mRNAs in females formed by exons 1–4. These differ in the length of their 3′UTR, yet they encode the same female Tra protein. In males, 5 distinct mRNAs (M1–M5) are produced depending on the male-specific exons included (see Fig. 1B). These latter exons contain translation stop codons. The comparison of male and female *Aotra* mRNAs indicates that they arise by sex-specific splicing of the *tra* pre-mRNA, with the male-specific exons being skipped in the female mRNA.

Figure 1A compares the molecular organisation of *Aotra, Cctra* and *Botra*. In all three cases the female mRNA contains 4 exons. Exons 2–4 are homologous across all three species, their sizes differing only slightly. Exon 1 of *Aotra* corresponds to exon 1 of *Cctra* and to exons 1A and 1B of *Botra*. In *Cctra* and *Aotra*, exons 1 and ms1 are contiguous. However, in *Botra*, exon 1 is split and exon 1A precedes and is contiguous with ms1. Both are separated by an intron from the downstream exon 1B with which exon ms2 is contiguous. This latter exon is flanked by introns in *Aotra* and *Cctra*. Both *Botra* and *Aotra* have an additional male-specific exon, ms3.

The conceptual translation of the female *Aotra* mRNA shows it to encode a polypeptide of 417 amino acids. Male *Aotra* mRNA, however, encodes a truncated, presumably non-functional polypeptide of either 55 or 67 amino acids depending on the male-specific splicing pathway followed (see Fig. 1B). The female Tra protein contains the SR dipeptides that characterise the family of SR proteins.

In *D. melanogaster* [26]], in other drosophilids [17], in *C. capitata* [18] and in *B. oleae* [19], the gene *tra* is closely linked to the well-conserved gene *l(3)73Ah*. In the three named species, these two genes are transcribed in opposite directions, and in *D. melanogaster* and *C. capitata* the genes *tra* and *l(3)73Ah* overlap at their 3′-UTR regions. In *B. oleae* such overlapping seems not to be present and the stop codons of both genes are 830 bp from each other. In *A. obliqua* the genes *tra* and *l(3)73Ah* do not overlap either, and are separated by about 3.5 kb (data not shown).

### The expression of *tra* in *A. obliqua*

**Figure 2:**
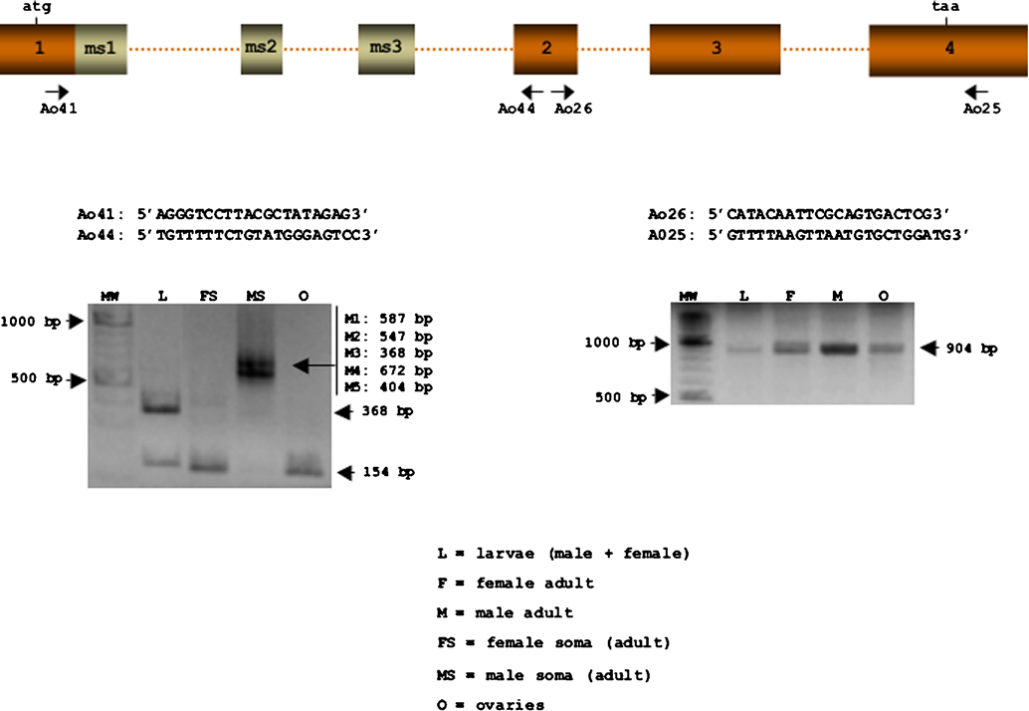
Expression of *A. obliqua tra*. RT-PCR analyses of total RNA from male plus female larvae (L), female adult (F), male adult (M), female soma (head plus thorax), male soma (head plus thorax), and ovaries (O). The sequence of the primers used and their locations are shown.

The expression of *Aotra* was studied by performing RT-PCR on total RNA from adult males and females, on RNA from the heads plus thoraces of male and female *A. obliqua* adults (separately), on that from a mixture of male plus female larvae at different developmental stages, and that from adult ovaries. Figure 2 shows the primers used. All the amplified fragments were cloned and sequenced. The primer pair Ao26 plus Ao25 amplified a fragment of 904 bp common to both sexes, while Ao41 plus Ao44 amplified a single fragment of 154 bp in adult female soma and ovaries, as well as two fragments of 154 and 368 bp in the larvae (corresponding to female and male mRNAs respectively). Different fragments were amplified in adult male soma depending on the male-specific exons they included (these latter fragments could not be resolved in gels).

The gene *tra* is transcribed in both sexes to produce two different spliced mRNAs−one in each sex−during development and adult life. In females, the mRNA encoding the full-length Tra protein is produced, whereas in males mRNA encoding a truncated, non-functional Tra protein is made. Importantly, *tra* is also expressed in the ovaries where it produces female mRNA. This indicates that, as in *C. capitata* and *B. oleae*, the mother provides the zygote with female *tra* mRNA and/or female Tra protein.

### The Tra protein of other *Anastrepha* species

The strategy followed to identify the *tra* ORFs in the other *Anastrepha* species is explained in Materials and Methods. The putative Tra proteins from the 12 *Anastrepha* species and the Tra protein from *C. capitata*, *B. oleae* and *A. obliqua* (used as reference for the genus *Anastrepha*) were then compared.

The Tra protein of the 12 *Anastrepha* species is composed of 417 amino acids, except that of *A. grandis*, which contains 416 amino acids (Fig. S1 in Supporting material). Their degree of similarity (i.e., identical plus conservative amino acids) ranges from 88 to 99% (upper half of Table 1). The Tra protein of the tephritids is larger than that of the drosophilids due to its bigger amino terminal end. This is composed of about 103 amino acids in the *Anastrepha* species and of 105 amino acids in *Ceratitis* and *Bactrocera*. The comparison of the specific amino-terminal region in all *Anastrepha* species revealed an extraordinary high degree of similarity (between 89–100%) (lower half of Table 1).

**Table 1:** Percentage of similarity among the *Anastrepha* Tra proteins.

species	*obl*	*sp1*	*sp2*	*sp3*	*sp4*	*grd*	*ser*	*sor*	*str*	*bis*	*ami*	*lud*
***obl***		**96**	**97**	**98**	**96**	**90**	**89**	**99**	**97**	**95**	**96**	**97**
***sp1***	*97*		**96**	**95**	**95**	**89**	**88**	**96**	**98**	**94**	**96**	**96**
***sp2***	*98*	*97*		**96**	**96**	**90**	**89**	**97**	**97**	**95**	**96**	**98**
***sp3***	*100*	*97*	*98*		**95**	**89**	**88**	**97**	**96**	**94**	**95**	**96**
***sp4***	*95*	*96*	*95*	*95*		**88**	**87**	**95**	**96**	**94**	**95**	**96**
***grd***	*90*	*91*	*90*	*90*	*89*		**89**	**90**	**90**	**91**	**90**	**89**
***ser***	*92*	*93*	*92*	*92*	*91*	*91*		**89**	**89**	**90**	**88**	**89**
***sor***	*100*	*97*	*98*	*100*	*95*	*90*	*92*		**97**	**95**	**96**	**97**
***str***	*98*	*99*	*98*	*98*	*97*	*92*	*94*	*98*		**95**	**97**	**97**
***bis***	*96*	*96*	*95*	*96*	*94*	*91*	*93*	*96*	*97*		**94**	**95**
***ami***	*96*	*97*	*96*	*96*	*95*	*94*	*94*	*96*	*98*	*97*		**96**
***lud***	*99*	*98*	*99*	*99*	*96*	*91*	*93*	*99*	*99*	*96*	*97*	

The upper half of the table shows the similarity values (in bold) for the entire Tra proteins; the bottom half shows the similarity values (in italic) for the specific amino terminal regions (the first 103 amino acids). *Anastrepha* species: obl, *A. obliqua;* sp1, *A.* sp.1 *aff*. *fraterculus*; sp2, *A.* sp.2 *aff*. *fraterculus*; sp3, *A.* sp.3 *aff*. *fraterculus*; sp4, *A.* sp.4 *aff*. *fraterculus*; grd, *A. grandis*; ser, *A. serpentina*; sor, *A. sororcula*; str, *A. striata*; bis, *A. bistrigata*; ami *A. amita*; lud, *A. ludens.*

**Figure 3:**
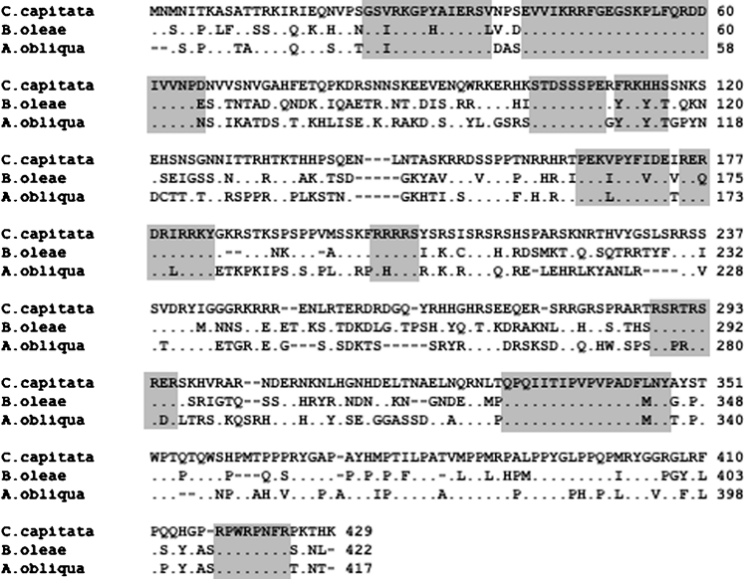
Comparison of the predicted Tra polypeptides of *C. capitata* [18], *B. oleae* [19] and *A. obliqua* (taken as the reference for the twelve *Anastrepha* species here studied). The shadowed regions correspond to the domains that show 100% similarity among the three species (similarity refers to identical plus conservative amino acids)

Table 2 and Fig. 3 compare the Tra protein of the tephritids *C. capitata, B. oleae* and *A. obliqua* (the reference for the genus *Anastrepha*). They differed slightly in the number of amino acids and the degree of similarity ranged between 54 and 56% (upper half of Table 2). A similar degree of conservation was seen when the specific amino-terminal region of the tephritid Tra protein were compared among the tephritid species (lower half of Table 2). In Fig. 3, the most conserved regions are shaded. Notice the two large domains in the amino-terminal end. Finally, the Tra proteins of the three tephritids *Anastrepha, Ceratitis* and *Bactrocera* contain SR dipeptides like the Tra proteins of the drosophilids, a feature that shows the SR proteins to be involved in splicing regulation. These SR dipeptides are also found in the specific amino terminal region of the three tephritids.

**Table 2:** Percentage of similarity among the tephritid Tra proteins.

Tephritid	*C. capitata*	*B. oleae*	*A. obliqua*
***C. capitata***		**56**	**54**
***B. oleae***	*61*		**54**
***A. obliqua***	*64*	*59*	

The upper half of the table shows the similarity values (in bold) corresponding to the entire Tra proteins of the Tephritids; the bottom half shows the values (in italic) for the specific amino terminal regions (the first 105 amino acids in *C. capitata* and *B. oleae*, and the first 103 amino acids in *A. obliqua*, the reference for the genus *Anastrepha*).

### Molecular organisation of the sex-specific splicing regulatory region of *tra*

The Tra protein acts as a splicing activator in the female-specific splicing of *dsx* pre-mRNA in *C. capitata* [18] and *B. oleae* [19]. In these species, the sex-specific splicing of *dsx* pre-mRNA is organised as in *Drosophila*: the male-splicing pathway represents the default mode, and the presence of functional Tra protein in females seems to cause the formation of the Tra-Tra2 complex, which binds to its targets in the female-specific exon, thus promoting the inclusion of the latter in the mature mRNA [11], [12], [13], [14], [15], [16]. The RBP1 being also needed [27]. The presence of Tra-Tra2 binding sites in the female-specific exon of *dsx* pre-mRNA of the *Anastrepha* species suggests that, in these species, Tra probably also controls the sex-specific splicing of *dsx* pre-mRNA [28], [29].

The auto-regulation model proposed by Pane et al. [18] for *C. capitata tra* consider that the Tra protein acts as a splicing inhibitor of its own pre-mRNA splicing (see Introduction). A similar model seems to be applicable to the other tephritids *Bactrocera* [19] and *Anastrepha* [this work] *tra* genes, since the molecular organisation of *tra* pre-mRNA of these tephritids were similar to that of *Ceratitis*. Nothing is presently known about the mechanism through which the tephritid Tra protein controls the splicing of its own transcript. We undertook a comparison of the *tra* genomic region (encompassing the male-specific exons and their flanking introns, where the regulation of sex-specific splicing occurs) of the twelve *Anastrepha* species studied here, of *C. capitata*, and of *B. oleae*. Given the possible involvement of Tra2 in the auto-regulation of *tra*, we also looked for the presence and location of putative Tra-Tra2 and RBP1 binding sites, as well as Tra2-ISS binding sites [30], in this *tra* genomic region. The *tra* genomic region corresponding to the *Anastrepha* species was amplified by PCR processing of genomic DNA using the primer pair TRA39 and TRA41 (see Materials and Methods and Table S1 in Supporting material).

**Figure 4:**
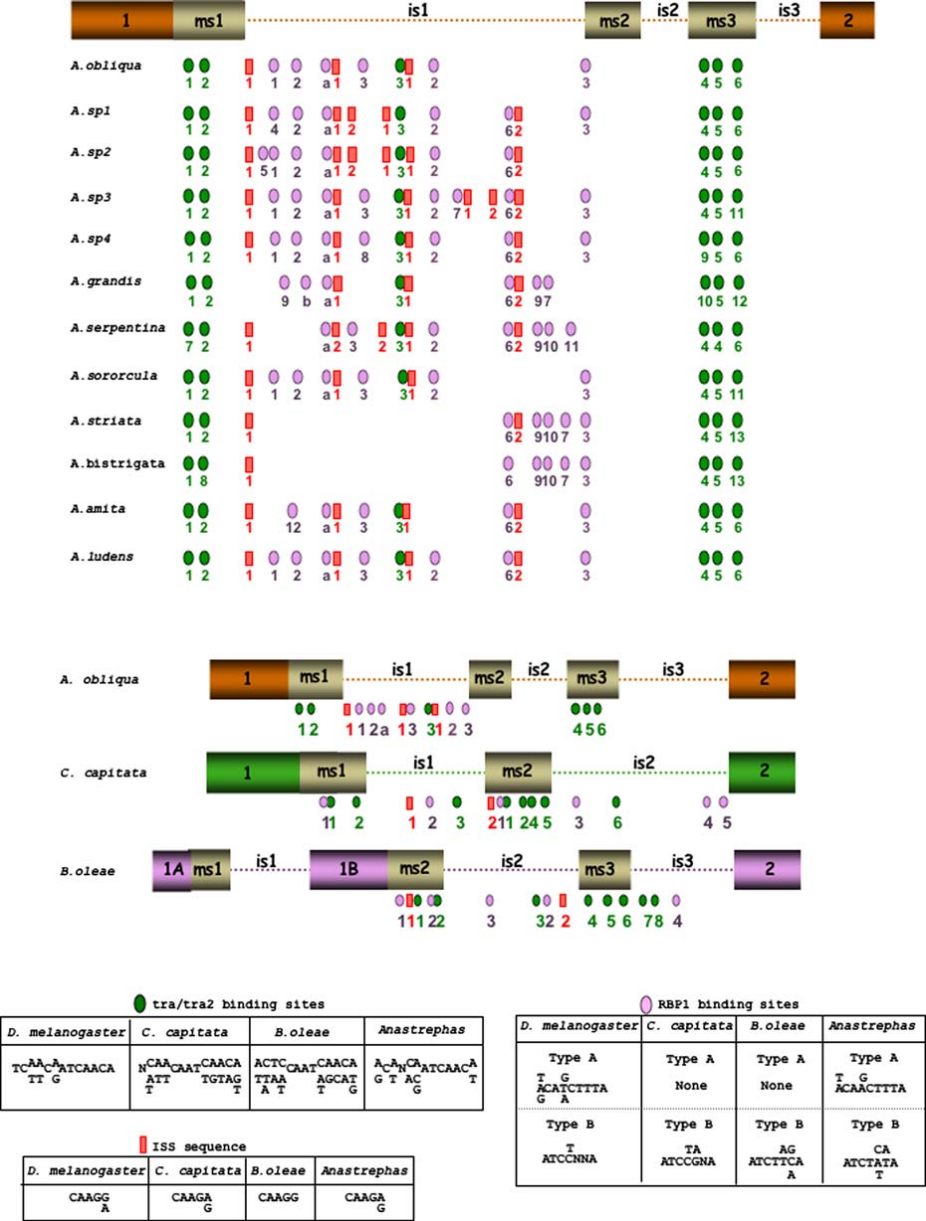
Comparison of the molecular organisation of the *tra* genomic region (encompassing the male-specific exons and their flanking introns) involved in sex-specific splicing regulation of the *tra* pre-mRNA, in the twelve *Anastrepha* species, in *C. capitata* (unpublished sequence) and in *B. oleae* (accession number AJ715414). Boxes represent exons, lines represent introns (not drawn to scale). In *B. oleae*, exon 1 is split into exons 1A and exon 1B [19]. The male-specific exons are denoted by ms1, ms2 and ms3, and the introns corresponding to the compared *tra* genomic region by is1, is2 and is3. The locations of the Tra-Tra2, RBP1 and Tra2-ISS binding sites are shown, along with their consensus sequences, together with those found in *D. melanogaster* (bottom of the Figure). The numbers and the letters (a) and (b) underneath the small rectangle and ellipsoids representing these binding sequences refer to the exact same sequences described in the Supporting Material.

**Figure 5:**
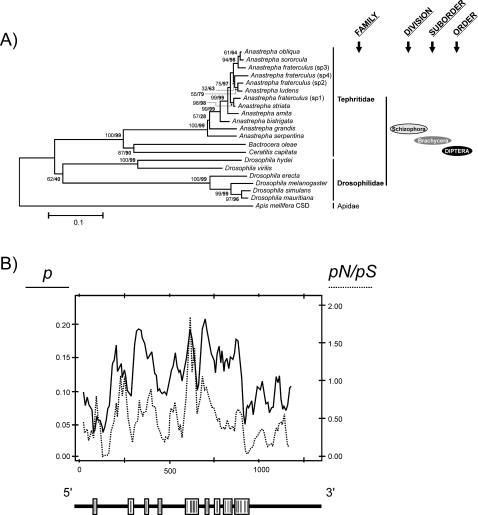
(A) Phylogenetic tree reconstructed from 22 protein TRA sequences belonging to different insect species (taxonomic groups indicated on the right hand side of the tree). The tree was built using the neighbour-joining method. The confidence levels for the groups are indicated in the corresponding nodes as bootstrap (BP, normal type) and interior branch test results (CP, bold type) based on 1000 replications. Values are shown only when either the BP or CP values are higher than 50%. (B) Proportion of nucleotide sites at which two sequences being compared are different (*p*, nucleotide substitutions per site) and ratio between the numbers of non-synonymous (*pN*) and synonymous (*pS*) substitutions per site across the coding regions of *tra* in the tephritids. These values were calculated using a sliding-window approach with a window length of 40 bp and a step size of 10 bp. The relative positions of the RS and SR dipeptides across *tra* are represented in the white boxes below the graph.

In the *Anastrepha* species, the size of intron is1 varied between 1024 and 3296 bp, and that of is2 between 337 and 472 bp. Intron is3 was composed of 85 bp in all twelve species (Fig. S2 in Supporting Material). Six putative Tra-Tra2 binding sites were found in all the *Anastrepha* species except for *A. striata* and *A. bistrigata*, which had five sites (Fig. 4). These elements were located at similar positions: two sites in exon ms1, one site in intron is1 (missing in *A. striata* and *A. bistrigata*), and three sites in exon ms3. Between 5 and 8 putative RBP1 binding sites were found, all located in intron is1. All the RBP1 binding sites were of type B but for two of type A (a and b in Fig. 4). Between one and six Tra2-ISS binding sites were found in intron is1 in all species (Fig. 5). The two CAAGG and CAAGA Tra2-ISS binding sequences reported in the *Drosophila tra-2* gene [30] were also found.

In *C. capitata*, eight repeats of Tra-Tra2 binding sites have previously been described [18]. In the present work, five type B RBP1 binding sites were recorded: one in exon ms1 and in exon ms2, one in intron is1, and three in intron is2 (Fig. 4). Finally, two types of Tra2-ISS binding sites were located, one in intron is1, and one in exon ms2 (Fig. 4).

In *B. oleae*, eight repeats of Tra-Tra2 binding sites have previously been reported [19]. Five type B RBP1 binding sites were seen in this work: two in exon ms2, another two in intron is2, and one in intron is3 (Fig. 4). Finally, two Tra2-ISS binding sites (both CAAGG-type) were identified; one in exon ms2 and the other in intron is2 (Fig. 4).

The comparison of the respective consensus sequences for all these binding sites in the *Anastrepha* species, *C. capitata* and *B. oleae*, revealed a high degree of similarity among the tephritids and *Drosophila* (Fig. 5). The complete sequences of all these binding sites are given in the Supporting Material (Fig. S3). Another shared feature is the mixing and grouping of these binding sites.

### Phylogeny and molecular evolution of gene *tra*

As in former studies of the Sxl [31] and Dsx [29] proteins, the topology of the phylogenetic relationship for Tra proteins from different tephritid and drosophilid species were in very good agreement with these species' taxonomic relationships. The different groups defined by the tree were very well supported by statistical tests (CP and BS). The tephritids and drosophilids grouped into distinct clades, and among the tephritids, *B. oleae* and *C. capitata*, which belong to the subfamily Dacinae, grouped together in a branch distinct from that encompassing the twelve species of *Anastrepha* (a genus that belongs to the subfamily Trypetinae). In the *Anastrepha* branch, species of the more ancient intrageneric taxonomic groups, such as *A. serpentina, A. grandis* and *A. bistrigata* (but not *A. striata*), lie in sub-branches distinct from those grouping the eight species belonging to the *fraterculus* taxonomic group.

Table 3 provides information showing the variation of the *tra* gene. Within the pool of three tephritid genera (*Anastrepha, Ceratitis* and *Bactrocera* species), the degree of amino acid variation is significantly smaller than that seen within the genus *Drosophila*. When variation of the Tra protein within the genus *Anastrepha* was evaluated, a still more reduced degree of Tra protein variation was found. The observed levels of protein variation agreed well with those observed at the nucleotide level, finding high levels of silent variation.

**Table 3:** Average number of amino acid and nucleotide differences per site among *tra* genes in different species, plus standard errors, calculated using the bootstrap method (1000 replicates).

	pAA (1)	pNT (2)	pS (3)	pN (4)	R (5)	Z-test	P-value
*Anastrepha*	0.056±0.006	0.034±0.002	0.049±0.005	0.028±0.003	1.5	3.68	0.000**
*Drosophila*	0.415±0.021	0.37±0.011	0.486±0.019	0.328±0.017	0.6	6.093	0.000**
Tephritidae	0.159±0.008	0.119±0.004	0.185±0.007	0.098±0.005	1.0	10.457	0.000**
Overall	0.452±0.012	0.379±0.006	0.488±0.011	0.345±0.01	0.6	9.361	0.000**

(1) Numbers of amino acid differences per site (p-distance). (2) Number of nucleotide differences per site (p-distance). (3) Number of synonymous differences per site. (4) Number of non-synonymous differences per site. (5) Transition/transversion ratio.

The Tra protein belongs to the family of SR-proteins. These are characterised by having serine-arginine (SR) dipeptides, which are involved in protein–protein interactions (reviewed in [32]). The percentage of SR dipeptides in the different species was determined, and means of 7.072% for the *Anastrepha* species and 5.644% for the other tephritid species *C. capitata* and *B. oleae* recorded. These values are low compared to the estimated 16.5% in *Drosophila* species [17]. Figure 5B shows the proportion of nucleotide sites at which two sequences being compared are different (*p*) against the ratio between the numbers of non-synonymous and synonymous nucleotide substitutions per site (*pN/pS*). The results show a high degree of coincidence between peaks of *p* and those of *pN/pS*, matching the gene regions encoding the SR domains, indicating a higher degree of nucleotide diversity in these particular areas.

## Discussion

### Sex determination in tephritid flies: the role of the *tra* gene

The present work shows that the gene *tra* of the *Anastrepha* species has a molecular organisation and expression pattern similar to those of *Ceratitis* [18] and *Bactrocera* [19]. The hypothesis of Pane et al. [18] regarding the role played by *tra* in *Ceratitis* sex determination−namely that of the memory device for sex determination through its auto-regulatory function–therefore applies not only to *Bactrocera* [19] but also to the *Anastrepha* species (this work), in which females are XX and males are XY. This hypothesis states that the Tra protein, together with the Tra2 protein, participate in the female-specific splicing of its own primary transcript. The maternal expression of *tra* would supply *tra* mRNA (or its protein) to the oocyte, thus making it available to the embryo. This would impose female-specific splicing on the initial zygotic *tra* pre-mRNA, which would give rise to the initial zygotic functional Tra protein and consequently the establishment of *tra* auto-regulation. Thus, XX embryos follow the female developmental route. However, XY embryos are able to follow the male route. For example, in male embryos of *C. capitata* it is known that the Y chromosome contains a male-determining factor (M factor) [33] that would prevent the instigation of *tra* auto-regulation. Consequently, these embryos would not produce functional Tra protein, leading to male development. In *Bactrocera* and *Anastrepha* it is still not known whether the Y chromosome is male-determining. However, the similar molecular organisation and expression pattern of *tra* in *Ceratitis* [18], *Bactrocera* [19] and *Anastrepha* (this work) suggest that the *tra* memory device mechanism, as well as the M factor mechanism for preventing the establishment of *tra* auto-regulation, might be present in all three of these extant genera, and that they may have been present in the common ancestor of the frugivorous Tephritidae lineages.

These results support the model of Wilkins [34], who proposed that the evolution of sex-determining cascades was bottom up, with the genes at the bottom being more conserved than those further upstream genes (for a theoretical analysis of this model see [35]). Indeed, the *tra/tra2>dsx* elements at the bottom of the cascade, and their relationships, have been found conserved in all the dipterans analysed so far. This suggests that they represent the ancestral state (which still exists in the Tephritidae and Muscidae lineages) with respect to the extant cascade found in the more evolved Drosophilidae lineage (in which *tra* is just another component of the sex determination gene cascade regulated by *Sxl*). Thus, in the phylogenetic lineage that gave rise to the drosophilids, evolution co-opted for the *Sxl* gene, modified it, and converted it into the key gene controlling sex determination, thus substituting for the loss of *tra* auto-regulation.

### The gene *tra* controls sex determination in the tephritid insects through a dual mechanism

The Tra protein in the tephritids *Ceratitis, Bactrocera* and *Anastrepha* appears to show a dual splicing role. On one hand it behaves as a splicing activator of *dsx* pre-mRNA−the binding of Tra to the female-specific exon promotes the inclusion of this exon into the mature mRNA. On the other hand, Tra acts as a splicing inhibitor of its own pre-mRNA−the binding of Tra to the male-specific exons prevents the inclusion of these exons into the mature mRNA. These observations raise the question of how Tra can perform this dual function. In this respect, the results obtained by other authors [30] with respect to *Drosophila* Tra2 and RBP1 function are pertinent here. The *Drosophila* Tra2 protein shows a dual splicing role. It behaves as a splicing activator of *dsx* pre-mRNA in the soma of *Drosophila* females, but also acts as a splicing inhibitor of the M1 intron in *tra-2* pre-mRNA in the germ line of *Drosophila* males. This inhibition is exerted through the binding of Tra2 to specific ISS sites. However, the *in vitro* interaction between Tra2 and its ISS targets is not sufficient to cause M1 splicing inhibition; the presence of nuclear extracts is also required, suggesting the existence of a yet unknown factor involved in the Tra2-ISS interaction [30]. This factor cannot be the Tra protein because this is not produced in *Drosophila* males (see Introduction). The RBP1 protein is also required for splicing inhibition of intron M1[30] in addition to being required for promoting the splicing of the female-specific exon of *dsx* pre-mRNA [27]. Thus, the dual role of Tra protein in the tephritids appears to parallel that of Tra2 and RBP1.

This prompted us to look for Tra2 ISS and RBP1 binding sites in the *tra* genomic region, which controls the sex-specific splicing of its primary transcript in *C. capitata*, *B. oleae* and the *Anastrepha* species. In addition to the previously described putative Tra-Tra2 binding sequences, putative Tra2-ISS and RBP1binding sites were found–an important discovery. These sequences are highly conserved in the tephritids and in *Drosophila*. Moreover, RBP1−but not Tra2-ISS−binding sites was found in the region of *Anastrepha*, *Ceratitis* and *Bactrocera dsx* pre-mRNA involved in sex-specific splicing regulation (data not shown). It is suggested here that the Tra2-ISS binding sites provide a discriminative feature for the *tra* and *dsx* pre-mRNAs regions involved in sex-specific splicing regulation.

Questions naturally arise regarding the molecular basis underlying the putative dual splicing role of the tephritid Tra protein. The presence and molecular organisation of Tra-Tra2 and RBP1 binding sites in the *dsx* gene of tephritid flies−which is similar to that seen in *Drosophila*−suggests that Tra, Tra2 and RBP1 bind co-operatively to form a splicing activator complex at the female-specific exon of *dsx* pre-mRNA. This would allow the exon to be incorporated into the mature mRNA. The tephritid *tra* pre-mRNA also contains Tra-Tra2 and RBP1 binding sites so that the Tra-Tra2-RBP1 complex can bind the male-specific exons−but in this case it would prevent the incorporation of these exons into the mature *tra* mRNA. It is proposed here that this is so because the presence of Tra2-ISS binding sites in the *tra* pre-mRNA determines the additional binding of Tra2 to these sequences, and that this overrides the activator effect of the Tra-Tra2-RBP1 complex.

**Figure 6:**
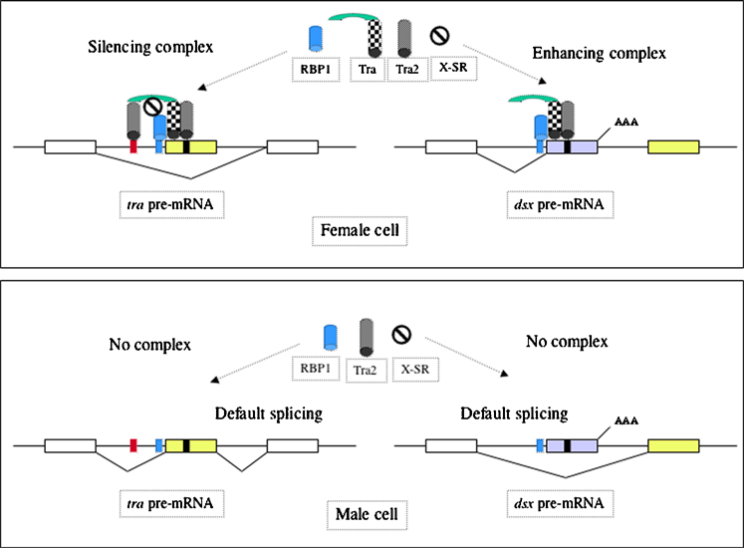
Proposed explanation of the dual role-played by the tephritid Tra protein in sex determination. For the sake of simplification, the *tra* pre-mRNA is shown as containing a single male-specific exon (yellow box); the white boxes represent common exons. The *dsx* pre-mRNA shows only the one common exon (white box), a female-specific exon (grey box) and a male-specific exon (yellow box). The lines represent introns. AAA stands for polyadenylation. The black, yellow and red rectangles represent the Tra-Tra2, the RBP1 and the Tra2-ISS binding sites respectively. The X-SR factor refers to the unknown factor mentioned in the text. The green part of the Tra protein corresponds to the amino terminal region of the tephritid Tra protein, which is not present in the Tra protein of the drosophilids. The complex made up by Tra, Tra2, RBP1 and X-SR inhibits splicing, whereas the complex formed by Tra, Tra2 and RBP1 promotes splicing. For details see text.

As mentioned above, the interaction of Tra2 with the ISS sites in *Drosophila tra-2* pre-mRNA requires an as yet unknown factor. Thus, it is possible that such a factor is also required for the interaction of Tra-2 at the ISS sites in tephritid *tra* pre-mRNA, and that the complex formed by Tra2 and the proposed factor blocks, either by itself or by recruiting another SR protein, the splicing activation effect of the Tra-Tra2-RBP1 complex. While in *Drosophila* the Tra protein cannot be this unknown factor, it cannot be ruled out that this is not the case in the tephritids. The tephritid Tra protein has an amino terminal region not found in the *Drosophila* Tra protein. This region is very strongly conserved among the tephritid *Ceratitis, Bactrocera* and *Anastrepha* species. In addition it contains SR-dipeptides involved in protein-protein interactions. Thus, the possibility exists that the tephritid Tra protein has a double interaction with the Tra2 protein−one mediated through the region of the protein homologous in the tephritids and drosophilids, and a second through the specific amino terminal region associated with the binding of Tra2 to ISS sites. This second interaction would convert the Tra-Tra2-RBP1 splicing activator complex into a Tra2-Tra-Tra2-RBP1 splicing inhibitor complex. Alternatively, the double interaction of Tra and Tra2 may recruit an SR protein, which would inhibit splicing. In this scenario, the amino terminal region of the tephritid Tra protein would be essential for this protein to exert its auto-regulation. Although both *tra* and *dsx* are transcribed within the same cell, and therefore their primary transcripts are exposed to the same splicing machinery, the splicing inhibitor complex (howsoever composed) would only be formed at *tra* pre-mRNA since only this contains Tra2-ISS sites (see a simplified diagram in Fig. 6).

Under this scenario, it is hypothesised here that in the phylogenetic lineage that gave rise to the drosophilids, the *tra* gene lost the Tra2-ISS binding sites and the Tra protein lost the amino terminal region that characterises the tephritid Tra protein; hence, its female-specific auto-regulatory function disappeared. This protein was, however, still feasible in the drosophilids because the *tra* gene gained Sxl-binding sequences so that the female-specific splicing regulation of the *tra* pre-mRNA came under the control of *Sxl*, which is only present in females.

### The molecular evolution of Tra

The phylogeny of *tra* reconstructed in the present work agrees well with the phylogenetic relationships among the tephritids and the drosophilids. The level of variation at both the nucleotide and amino acid level differs between the drosophilids and the tephritids. This might be due to the protein's auto-regulatory function in the latter.

Particularly interesting is the comparison of *tra* variation between the *Drosophila* and *Anastrepha* species. McAllister and McVean [36] reported high rates of neutral evolution when comparing *D. americana* and *D. virilis* (separated 60 Myr ago), while Kulathinal et al. [37] reported a high level of divergence among the sibling species of the Melanogaster complex *D. melanogaster, D. simulans, D. mauritiana* and *D. sechellia* (separated 2.5 Myr ago). In this work, the *tra* gene of the twelve *Anastrepha* species showed extraordinarily reduced variation, and no insertions were detected, unlike in some *Drosophila* species. Indeed, silent variation appears to be significantly more common than non-silent variation when considering the complete coding region of *tra*, suggesting that this gene is subject to strong purifying selection in order to preserve the mechanism of action of Tra proteins.

Although the SR dipeptide content of the tephritids and the drosophilids is also different, the distribution of these regions over the Tra protein is very much conserved within both groups. This supports the proposal that during the evolution the Tra proteins these maintained enough SR dipeptides regions to bestow functionality on these proteins [32]. The Tra proteins seem to lack an RNA binding domain so that its role in splicing regulation is exerted at the level of its interaction (through their SR domains) with other proteins carrying RNA-binding domains, such as Transformer-2 (reviewed in [32]).

## Materials and Methods

### Species

The species of *Anastrepha* studied, their host fruits, and the sites where they were collected are described in Ruiz et al. [29]. *Anastrepha ludens* was provided by Pablo Montoya (Programa Moscamed, Direccion General de Sanidad Vegetal, SAGAR, Apartado Postal 368, 30700 Tapachula, Chiapas, Mexico).

### Extraction of DNA and RNA

Total genomic DNA was isolated from flies according to Maniatis et al. [38] Total RNA extracts from frozen adult males and females were prepared using the Ultraspec-II RNA isolation kit (Biotecx) following the manufacturer's instructions. The GenomeWalker genomic library of *A. obliqua* was synthesised using the BD GenomeWalker Universal kit (BD Biosciences), following the manufacturer's instructions

### PCR and RT-PCR analyses

Five hundred nanograms of genomic DNA from each adult insect were used in PCR analyses. Five micrograms of total RNA from each were reverse transcribed with Superscript (Invitrogen) following the manufacturer's instructions. Ten percent of the synthesized cDNA was amplified by PCR. PCR and RT-PCR products were analysed by electrophoresis in agarose gels and the amplified fragments sub-cloned using the TOPO TA-cloning kit (Invitrogen) following the manufacturer's instructions. These subclones were then sequenced using universal forward and reverse primers. Figure 1 shows the sequences and location of the primers. Reverse transcription reactions were performed with the oligo-dT primer. The *tra* genomic region that is involved in sex-specific splicing regulation in all *Anastrepha* species was amplified using the pair of primers TRA39 and TRA41, which are common to all *Anastrepha* species. The sequences of all primers used in this work are shown in Table S1 in Supporting material.

### Isolation of gene *tra* of *A. obliqua*

The first step in the isolation of the *A. obliqua tra* gene (*Aotra)* was to perform RT-PCR on total RNA from female adults. Reverse transcription was performed using the primer oligo-dT, while PCR was performed with three designed primers: DOMA2+ and CcCATs-, specific for the beginning of exon 2 and close to the end of exon 4 of the *C. capitata tra (Cctra)* gene respectively, and PyA-, a degenerate primer designed after comparison of the *Cctra* and *B. oleae tra (Botra)* sequences. The two amplified fragments were cloned and sequenced. The conceptual amino acid sequences of these fragments showed a high degree of similarity with the region of the CcTra protein encoded by exons 2–4, indicating that a fragment of the putative AoTra protein had been isolated.

To determine the molecular organisation of *Aotra* the following strategy was followed. Firstly, 3′- and 5′-RACE analyses were performed. To this end, specific primers from the amplified sequenced were synthesised: AZ1+ and AZ2+ for 3′-RACE, and B41- and B42- for 5′-RACE. These primers were used in nested PCR reactions, the products of which were cloned and sequenced. The 5′-RACE generated three overlapping fragments of about 300, 450 and 500 bp. The largest one contained the start ATG codon of the *tra* ORF. Thus, exon 1 and the 5′UTR were identified. The 3′-RACE, which produced three overlapping fragments of about 243, 288 and 528 bp, allowed the identification of the end of exon 4 and the 3′UTR region containing the three poly-A(+) signals corresponding to the three amplified fragments. Consequently, the gene *Aotra* encodes three female mRNAs of 1894, 1654 and 1603 bp that differ in the length of the 3′-UTR depending on the poly-A(+) signal used. Next, RACE overlapping PCR was performed on cDNA synthesised from total RNA of adult males. The amplified fragments were cloned and their sequences compared to that of the female cDNA. Five different isoforms of male mRNA of 2326 (M1), 2285 (M2), 2108 (M3), 2412 (M4), and 2145 (M5) bp were found, depending on the male-specific exons included (see Fig. 1B).

Secondly, the GenomeWalker kit was used to perform PCR on genomic DNA of *A. obliqua* in order to determine the exon/intron junctions via genomic walking. The sequences of the genomic fragments thus generated were compared with the *A. obliqua* male and female cDNA sequences previously determined. In this way, the exon/intron junctions were unambiguously identified.

For identification of the Tra protein of other Anastrepha species, RT-PCR analyses of total RNA from female adults were performed. Reverse transcription was performed with the oligo-dT primer. PCR amplification of the cDNA was undertaken using the pair of primers TRA23 (Table S1) plus Ao25 (Fig. 2) corresponding to sequences of the Aotra gene. The first primer represents part of the exon 1 sequence, while the second represents part of the exon 4 sequence – respectively before and after the start and stop codons of the ORF. Thus, the amplicon expands the whole ORF. All amplicons were cloned in the TOPO-TA vector and subsequently sequenced.

### DNA sequencing

Sequencing was performed using an automated 377 DNA sequencer (Applied Biosystems). The following list shows the accession numbers for the *tra* gene of the *Anastrepha* species studied: *A. obliqua* (EU024498); *A.* sp.1 *aff*. *fraterculus* (EU024499); *A.sp2 aff fraterculus* (EU024500); *A. sp3 aff fraterculus* (EU024501); *A. sp4 aff fraterculus* (EU024502); *A. grandis* (EU024503); *A. serpentina* (EU024504); *A. sororcula* (EU024505); *A striata* (EU024506); *A. bistrigata* (EU024507); *A amita* (EU024508); and *A. ludens* (EU024509).

### Comparison of DNA and protein sequences

All comparisons were made using Fasta v.3.0t82 [39] and ClustalW1.83 software [40].

### Molecular evolutionary analyses

For comparison of DNA and protein sequences, and for phylogenetic analyses of gene *tra*, the methodology used for the analysis of genes *Sex-lethal* [31] and *dsx* [29] was followed. The analysis of nucleotide variation across coding regions was performed using a sliding-window approach, estimating the proportion (*p*) of nucleotide sites at which two sequences being compared are different, and the ratio between the numbers of non-synonymous (*pN*) and synonymous (*pS*) substitutions per site, with a window length of 40 bp and a step size of 10 bp. Estimates of the dipeptide compositions of TRA proteins from different dipteran species were made using the services of the COPid Server (http://www.imtech.res.in/raghava/COPid/index.html).

## Supporting Information

Table S1 (0.47 MB DOC)

Figure S1 Comparison of the predicted Tra polypeptides of the *Anastrepha* species. Points stand for the same amino acid. obl, *A. obliqua*; sp1, A. sp.1 *aff. fraterculus*; sp.2, A. sp.2 aff. fraterculus; sp.3, A. sp.3 *aff. fraterculus*; sp.4, A. sp.4 *aff. fraterculus*; grd, *A. grandis*; ser, *A. serpentina*; sor, *A. sororcula*; str, *A. striata*; bis, *A. bistrigata*; ami, *A. amita* and lud, *A. ludens*.
(0.15 MB TIF)

Figure S2 The genomic *tra* region of the *Anastrepha* species involved in sex-specific splicing regulation. The number of base pairs in the exons and introns is indicated. For the rest of the symbols see legend to Figure 1.
(0.14 MB TIF)

Figure S3 Sequence of the different putative Tra-Tra2, RBP1 and Tra2-ISS binding sites. The numbers in front of each sequence correspond to the numbers for these binding sites in Figure 5.
(0.12 MB TIF)
