# Supplementary material for: The Gene Transformer of Anastrepha Fruit Flies (Diptera, Tephritidae) and Its Evolution in Insects
Source: PLoS One. 2007 Nov 28;2(11):e1239. doi: 10.1371/journal.pone.0001239 (PMC2080774; doi:10.1371/journal.pone.0001239)
Supplement: Table S1 — (0.47 MB DOC) [file pone.0001239.s001.doc]

**Table S1.** Sequences of primers used in this work.

| **Primer** | **Sequence** |
| --- | --- |
| AZ1+ | 5’ TTCCAGTACCAGTACCTGC 3’ |
| AZ2+ | 5’ GCTGCCTATCACATACCAAC 3’ |
| B41- | 5’ CTGGACTGCTAGAATCTGTC 3’ |
| B42- | 5’ TTGGTTCGTGGAGGCGATC 3’ |
| CcCATS- | 5’ ATCCACGTCCACCGTAACG 3’ |
| DOMA2+ | 5’ AAACGTAGATTTGGTGAAGG 3’ |
| GWTRA11 | 5’ GATGTTTTTCTGTATGGGAGTCCGTAG 3’ |
| GWTRA16 | 5’ AGCTGCTGGTAAAATGGTTGGTATGAG 3’ |
| GWTRA20 | 5’ CCTCATACCAACCATTTTACCAGCAG 3’ |
| PYA- | 5’ ATYTCRTCIAYRAARTAIGG 3’ |
| TRAM1 | 5’ GCGGTTGAATTTATATGCGTG 3’ |
| TRA3 | 5’ CAACCGAGTATGTTTTCGTCC 3’ |
| TRA23 | 5’ CAACCAAACAGACAGCCGTC 3’ |
| TRA28 | 5’ CACTTTCAACGCAACACGAC 3’ |
| TRA29 | 5’ TGCAACTGGATGAATCAACAG 3’ |
| TRA30 | 5’ GGGTTCTCAACAATGTCATC 3’ |
| TRA39 | 5’ TCACGTTCTCTTGTCTCGTC 3’ |
| TRA41 | 5’ AGGGTCCTTACGCTATAGAG 3’ |
| TRA47 | 5’ ACCTTTATTTCAACGTGATGAC 3’ |
| TRA48 | 5’ TACTGGAATGGTTATTATCTGC 3’ |
