# Supplementary figures and images for: The Gene Transformer of Anastrepha Fruit Flies (Diptera, Tephritidae) and Its Evolution in Insects
Source: PLoS One. 2007 Nov 28;2(11):e1239. doi: 10.1371/journal.pone.0001239 (PMC2080774; doi:10.1371/journal.pone.0001239)

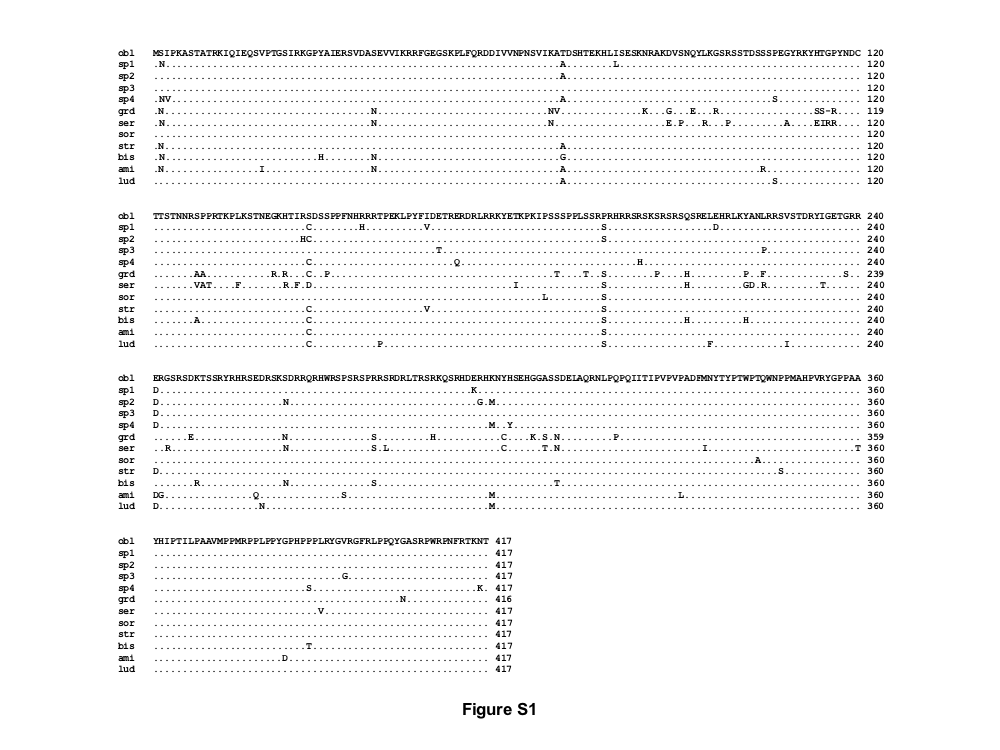

Supplement: Figure S1 — Comparison of the predicted Tra polypeptides of the Anastrepha species. Points stand for the same amino acid. obl, A. obliqua; sp1, A. sp.1 aff. fraterculus; sp.2, A. sp.2 aff. fraterculus; sp.3, A. sp.3 aff. fraterculus; sp.4, A. sp.4 aff. fraterculus; grd, A. grandis; ser, A. serpentina; sor, A. sororcula; str, A. striata; bis, A. bistrigata; ami, A. amita and lud, A. ludens. (0.15 MB TIF) [file pone.0001239.s002.tif]

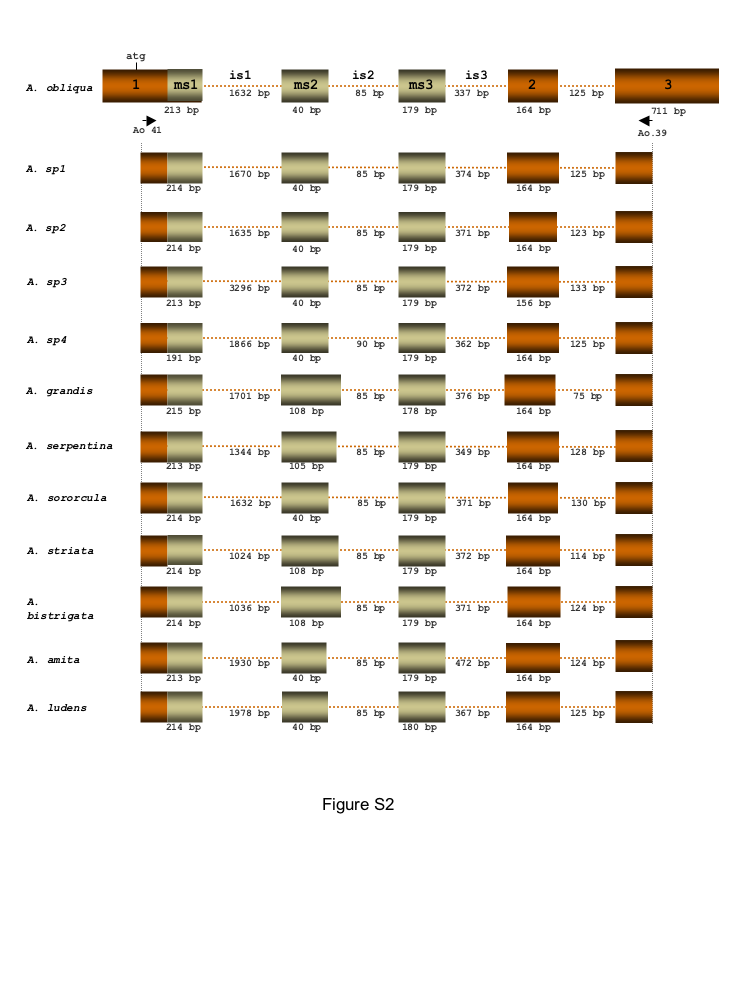

Supplement: Figure S2 — The genomic tra region of the Anastrepha species involved in sex-specific splicing regulation. The number of base pairs in the exons and introns is indicated. For the rest of the symbols see legend to Figure 1. (0.14 MB TIF) [file pone.0001239.s003.tif]

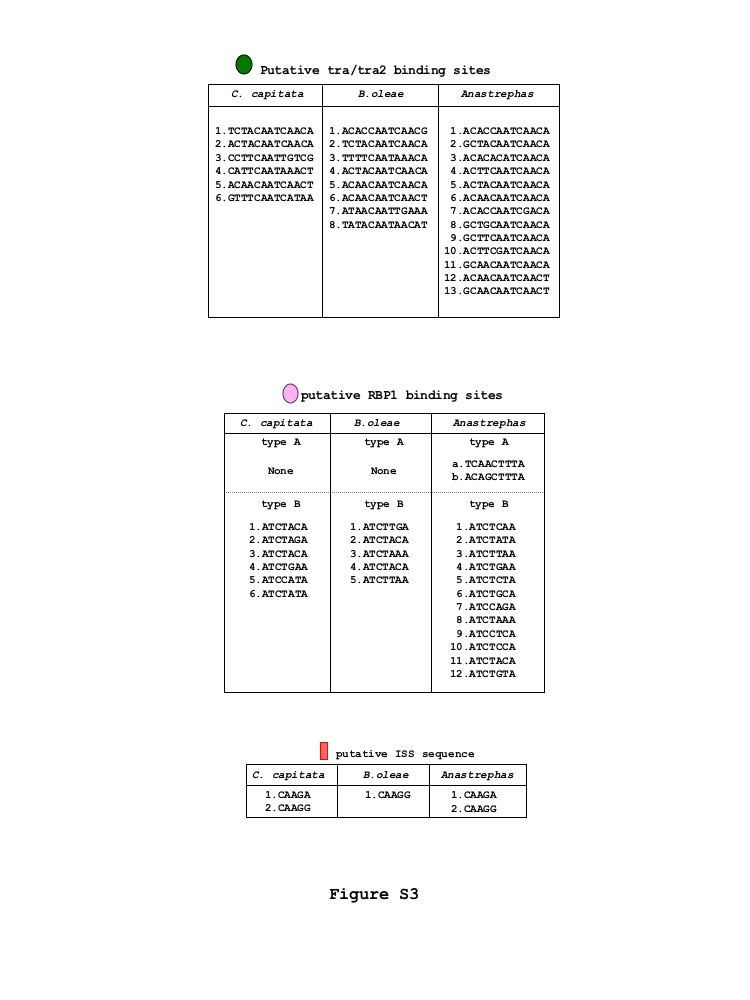

Supplement: Figure S3 — Sequence of the different putative Tra-Tra2, RBP1 and Tra2-ISS binding sites. The numbers in front of each sequence correspond to the numbers for these binding sites in Figure 5. (0.12 MB TIF) [file pone.0001239.s004.tif]
